# Evaluation of inhibitory control and attentional bias through eye-tracking: A modified emotional stop-signal task

**DOI:** 10.1016/j.mex.2025.103439

**Published:** 2025-06-13

**Authors:** Gonçalo Barros, Filipa Ribeiro

**Affiliations:** Universidade Católica Portuguesa, Faculty of Health Sciences and Nursing, CIIS, Portugal

**Keywords:** Obsessive-compulsive disorder, Inhibitory control, Emotional stop-signal task, Attentional biases, Eye-tracking, Cognitive markers, Modified Emotional Stop-Signal Task (MESST)

## Abstract

Obsessive-compulsive disorder (OCD) is characterized by deficits in inhibitory control and attentional processes. The emotional nature of stimuli can significantly influence these cognitive processes, yet traditional paradigms assessing inhibitory responses, such as the Stop-Signal Task, typically neglect emotional stimuli. This limitation reduces their capacity to capture the cognitive impairments associated with OCD fully. To address this gap, we introduce the Modified Emotional Stop-Signal Task (MESST), a novel paradigm designed to concurrently evaluate inhibitory control and attentional biases through eye-tracking technology. MESST integrates emotionally evocative stimuli into a standard stop-signal framework, allowing simultaneous measurement of Stop-Signal Reaction Time (SSRT) and attentional metrics such as latency to first fixation and total dwell time. Additionally, participants complete validated psychological scales—the State-Trait Anxiety Inventory (STAI), Barratt Impulsiveness Scale (BIS-11), and Obsessive-Compulsive Inventory–Revised (OCI-R)—providing detailed characterization of impulsivity and anxiety traits. Suitable for normative and clinical populations, MESST facilitates the investigation of interactions between emotional processing, cognitive control, and attentional biases, thereby advancing our understanding of the cognitive-emotional mechanisms underlying OCD and related disorders.

• Integrates emotional stimuli into a standard inhibitory control paradigm.
• Measures attentional processes concurrently via high-frequency eye-tracking.
• Applicable to both clinical and non-clinical populations.

## Graphical abstract

**Figure:**
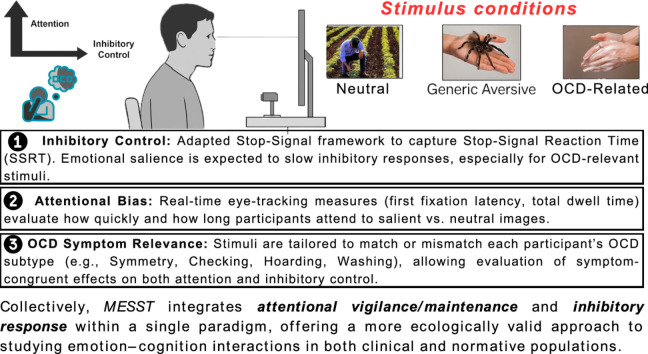

Specifications table

**Table:** 

Subject area:	Neuroscience
More specific subject area:	Cognitive & Behavioral Neuroscience
Name of your method:	Modified Emotional Stop-Signal Task (MESST)
Name and reference of original method:	Logan, G.D., Cowan, W.B., Davis, K.A., 1984. On the ability to inhibit simple and choice reaction time responses: A model and a method. Journal of Experimental Psychology. Human Perception and Performance 10, 276–291. https://doi.org/10.1037//0096–1523.10.2.276.
Resource availability:	*Task development:* SR Research *Experiment Builder* v2.3; full .*ebz* project and stimulus set in open repository https://github.com/eLbARROS13/MESST_Files. *Data Pre-processing:* SR Research *Data Viewer* for gaze parsing (same GitHub) *Data Analysis:* Python 3.11 scripts and SPSS v30, all provided in the repository. *Eye-tracking Equipment:* EyeLink Portable Duo (SR Research) *Response Device:* MilliKey MH 5 two button USB box *Sound Playback:* PreSonus *AudioBox USB* interface + Sennheiser HD series headphones

## Background

Obsessive Compulsive Disorder (OCD) provides a natural model for studying how emotional salience interferes with two core cognitive domains—inhibitory control and attention. Robust evidence shows that OCD patients exhibit prolonged Stop Signal reaction times (SSRTs) –a metric that describes inhibition impairments– and exaggerated gaze allocation toward threat-related cues, both phenomena linked to dysfunction in cortico-striato-thalamo-cortical circuitry [1,2]. Conventional Stop Signal Tasks (SSTs) quantify motor inhibition with millisecond precision [3,4], whereas dot-probe or Emotional Stroop paradigms index attentional bias indirectly via reaction time [5]. Crucially, these tasks are almost always administered **in isolation** and rely on emotionally neutral or standard stimuli, undermining ecological validity and masking the dynamic interplay between attention and inhibition that characterizes everyday compulsive behavior [6,7].

Recent adaptations—termed Emotional SSTs—integrate negative emotional stimuli within the standard SST framework and have provided evidence that threat-related content lengthens SSRTs [8]. However, two critical gaps remain. Firstly, current Emotional SST paradigms rarely tailor stimuli to the individual’s specific obsessional themes (e.g., contamination, symmetry), despite robust evidence indicating that personalized cues elicit stronger autonomic and neural responses in individuals with OCD [9]. Secondly, these paradigms seldom incorporate eye-tracking methods, thus leaving unclear whether impaired inhibitory performance results primarily from heightened attentional engagement or from motor inhibition deficits per se. Eye-tracking technology offers a continuous, high-resolution measure of gaze behavior, providing precise data regarding the timing (latency to first fixation—indicative of **attentional vigilance**) and duration (total dwell time—indicative of **attentional maintenance**) of visual engagement with stimuli. Importantly, eye-tracking ensures ecological validity by directly assessing attentional processes through gaze patterns, independently of secondary motor responses [10].

The **Modified Emotional Stop-Signal Task (MESST)** was engineered to address these limitations. MESST (i) randomizes neutral, generic aversive, and participant specific OCD congruent images across trials; (ii) applies an adaptive staircase to stabilize stop success at ≈50 %, ensuring reliable SSRT estimation; and (iii) synchronizes 2000 Hz eye-tracking with behavioral logging, thereby co-registering gaze dynamics and stopping on a single trial basis. By uniting these elements, MESST produces a rich multimodal dataset that can disentangle whether emotional interference acts primarily via attentional capture, inhibitory slowing, or their interaction.

Providing a transparent, step-by-step account of MESST will allow other researchers to (a) replicate and extend our findings in clinical or developmental cohorts; (b) customize the stimulus sets to probe disorder-specific triggers in anxiety, PTSD or addiction; and (c) integrate open-source analysis scripts for rapid hypothesis testing. Ultimately, MESST offers a scalable method for quantifying how emotionally salient information competes with top-down control—a process central to compulsive psychopathology and to theoretical models of emotion–cognition interaction.

## Method details

### Participants and screening

MESST was devised for two independent groups: (i) a healthy sample and (ii) a clinically diagnosed OCD sample. We estimated a sample size of 20–30 subjects per group. The required sample size was estimated using G*Power 3.1 software, based on a mixed-design ANOVA focusing specifically on the within-between interaction effect (image category × group). The following parameters were applied: anticipated moderate effect size (*f* = 0.25), corresponding to a partial eta-squared (η²ₚ) of ∼0.06. This estimate was based on prior SST literature, where emotional interference and group effects often yield small-to-moderate effects (η²ₚ ≈ 0.03–0.08; *d* ≈ 0.45–0.65) [7], making it a realistic and conservative target for the present mixed-design ANOVA. Statistical significance level (α = 0.05), and desired statistical power (1-β = 0.95). Considering a mixed design with 2 groups (normative and OCD), 6 repeated measures (stimulus categories), an assumed correlation among repeated measures of *r* = 0.50, and no correction for non-sphericity (ε = 1), the calculated sample size yielded a total of 28 participants (14 per group).

However, acknowledging potential attrition and typical data loss in eye-tracking paradigms (∼15–20 %), and considering more conservative scenarios (smaller effect sizes or lower correlations among repeated measures), we adjusted this estimate upwards. Consequently, our target recruitment range was set between 20 and 30 participants per group (total *N* = 40–60), ensuring adequate statistical power even under less optimal circumstances or assumptions.

**Healthy adults** (50 % Male, *N* = 20–25) are recruited through university mailing lists and screened online for handedness, normal or corrected to normal vision, and absence of neurological or psychiatric history. To meet the inclusion/exclusion criteria each prospective participant completes the *Hospital Anxiety and Depression Scale* (HADS) [11] and the Matrix Reasoning subtest of the Wechsler Adult Intelligence Scale-III (WAIS-III) [12] subtest at the start of the lab visit; only scores within normative limits are accepted (HADS<16; WAIS-III>7).

**OCD participants** (50 % Male, *N* = 20–25) are referred by clinicians or self–enroll from OCD support networks. Inclusion hinges on a primary DSM-5 OCD diagnosis verified by a psychiatrist and quantified with the *Yale -Brown Obsessive -Compulsive Scale* [13] (Y-BOCS; score ≥ 16). Comorbid psychosis, substance misuse or neurological disorders are exclusionary (Table 1).

**Table 1:** Recruitment and screening instruments.

Sample	Target N	Key inclusion	Primary screening	Exclusion
Healthy	20–30	Age ≥ 18, right-handed, normal vision	HADS, WAIS-III Matrix Reasoning	Neurol./psych. history
OCD	20–30	Same as healthy, DSM-5 OCD, Y-BOCS ≥ 16	Y-BOCS clinical interview, OCI-R	Comorbidity

Immediately before MESST, **all** participants complete the *State-Trait Anxiety Inventory* (STAIY1/Y2) [14], the *Barrat Impulsiveness Scale* (BIS11) [15] and the *Obsessive-Compulsive Inventory* (OCIR) [16]. STAIY1 is repeated post-task to detect any transient anxiety elicited by the emotional images. Ethical approval was granted by the Faculty of Health Sciences and Nursing Ethics Committee; written informed consent and withdrawal rights are emphasized, and a debrief follows the session.

### Apparatus and laboratory environment

**Fig. 1:**
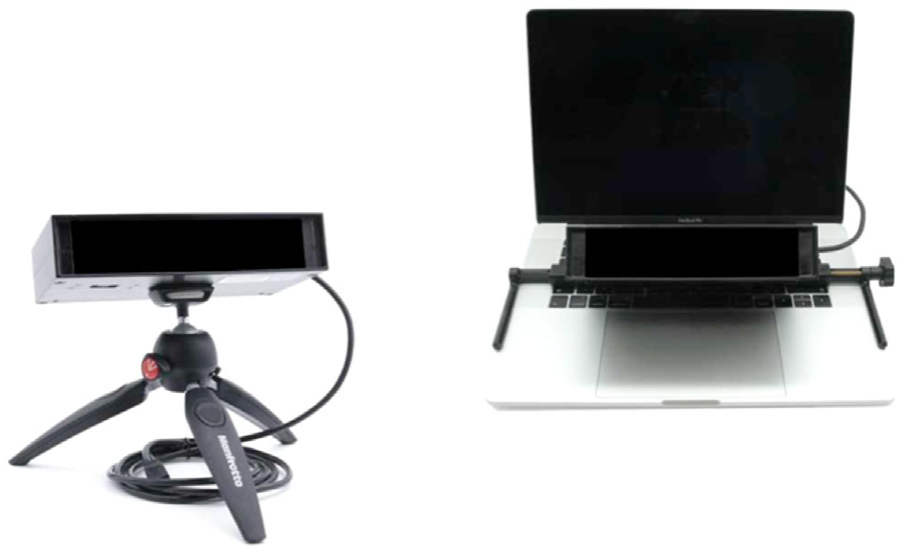
Eye-tracking system (EyeLink Portable Duo) used in the MESST paradigm.

MESST requires a dual-computer architecture to guarantee synchrony between stimulus delivery and gaze recording. The **host PC** runs the *EyeLink Host application* and communicates via gigabit Ethernet with a **display PC** that drives a 22-inch, 60 Hz LCD positioned 60 cm from the participant. Eye movements are recorded monocularly at 2000 Hz with an **EyeLink Portable Duo**, mounted below the display (Fig. 1). A **MilliKey MH-5** USB box registers the two-choice valence response; auditory stop-tones (750 Hz, 100 ms) are rendered through **Sennheiser HD-series** headphones connected to a *PreSonus AudioBox* USB sound card, ensuring <2 ms output latency.

The lab is dimmed to <5 lux and acoustically insulated (ambient noise <40 dB). Prior to each block, a nine-point calibration aims for <0.5° average error; drift correction is invoked whenever error exceeds 1° Table 2 lists hardware specifications.

**Table 2:** Hardware and software specifications.

Module	Specification
Eye tracker	EyeLink Portable Duo, 2000 Hz
Stimulus PC	22 ″ LCD, 1920 × 1080 @ 60 Hz
Control PC	EyeLink Host application on Windows 10
Response device	MilliKey MH-5 USB
Audio	750 Hz tone via Sennheiser HD-series, PreSonus AudioBox
Sync	Gigabit crossover cable

### Stimulus

To evoke condition-specific emotional interference, MESST deploys **six balanced image categories**: (1) neutral, (2) generic aversive, and four OCD congruent subsets (symmetry, checking, hoarding, washing). Neutral and generic aversive pictures are drawn from the *International Affective Picture System* (IAPS) [17] using specific valence/arousal scores for each category, in accordance with previous Emotional Paradigms [18], whereas OCD picture sets come from the *Maudsley Obsessive Compulsive Stimulus Set* (MOCSS) [9]. Every image is 800 × 600 px, luminance-matched and presented 8° left or right of center on a uniform grey background. Each 100trial block contains ∼16–17 images per category, preventing expectancy effects. Table 3 gives the valence/arousal profile.

**Table 3:** Stimulus categories.

Category	Source	Valence (mean)	Arousal (mean)
Neutral	IAPS	≈ 5.0 ± 1.0	≈ 2.8 ± 1.0
Generic aversive	IAPS	≈ 2.4 ± 1.0	≈ 6.1 ± 1.0
OCD-Symmetry	MOCSS	n/a	n/a
OCD-Checking	MOCSS	n/a	n/a
OCD-Hoarding	MOCSS	n/a	n/a
OCD-Washing	MOCSS	n/a	n/a

The MOCSS images used in this study were selected based on their symptom-specific relevance rather than generalized normative valence or arousal ratings. Specifically, clinicians with expertise in OCD first identified real-world objects and scenarios commonly reported by patients as provoking significant anxiety and compulsive urges, across four major symptom dimensions: contamination/washing, checking, hoarding, and symmetry/order. Digital photographs of these items—such as public toilets, open doors, chaotic rooms, and old newspapers—were taken to visually represent these symptom themes. These candidate images then underwent an initial screening by independent patients and healthy volunteers, who rated them on visual complexity, as well as levels of evoked anxiety and disgust. Images that were too visually simplistic or overly complex were excluded to ensure clarity and uniformity.

### Experimental design

The full task comprises **600 trials**, arranged as six blocks with brief self-paced breaks. Participants indicate whether the picture is *negative* or *nonnegative* by pressing the assigned left/right key, this response is based on how they perceive the emotional content of the image; on 25 % of trials a tone instructs them to **withhold** the response. An adaptive staircase controls the stop-signal delay (SSD): starting at 250 ms, SSD increases by 50 ms after each successful inhibition and decreases by 50 ms after a failed stop, converging on ≈50 % success [4].

A typical trial unfolds as follows:

1. Central fixation enforced for ≥200 ms (gaze-contingent).
2. Image appears randomly left/right; response window 0–1500 ms.
3. On Stop trials, the tone sounds after the current SSD.
4. Inter-trial interval = 1000 ms with central cross; next trial begins only after gaze re-enters the fixation ROI.

**Fig. 2:**
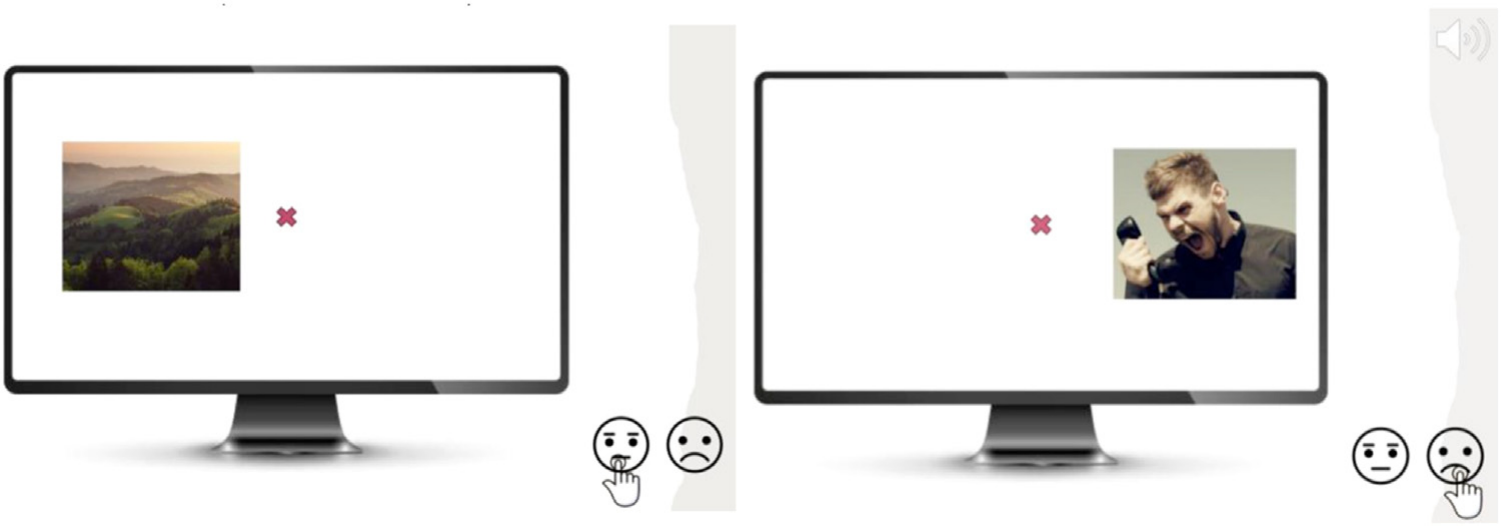
Example trials in MESST. Left: A Go trial with a neutral image is presented, and the participant must press the “non-negative” key. Right: A Stop trial with an aversive image is presented; an auditory stop-signal sounds shortly after image onset, and the participant must inhibit the response (not press the key).

No trial wise feedback is provided, preventing strategic slowing. Fig. 2 illustrates Go versus Stop trials.

### Data streams and derived variables

*Behavioral* data include Go trial reaction time (RT), classification accuracy, Stop trial outcome and all SSD adjustments. For each emotional category, **SSRT** is computed with the integration method^3^:

$$SSRT=RT_{p}-\;\bar{SSD}$$

where $RT_{p}$ is the p-th percentile of the correct-Go RT distribution, with p equal to the individual’s Stop-failure rate.

*Ocular* data are parsed in *Data Viewer* with stimulus-specific areas of interest (AOIs). Principal metrics are (i) **latency to first fixation** on the picture AOI (attentional vigilance), (ii) **total dwell time** within that AOI (maintenance), and (iii) fixation count. Trials lacking a stimulus fixation or with >25 % sample loss are dropped from ocular analyses.

All behavioral and eye-tracking timestamps share a common clock, allowing single-trial fusion and time locked visualizations.

### Statistical workflow

All statistical analyses are conducted using Python 3.11 and IBM SPSS Statistics v30. Prior to inferential testing, assumptions of normality (Shapiro–Wilk test) and sphericity (Mauchly’s test) are verified. When sphericity is violated (ε < 0.75), Greenhouse–Geisser corrections are applied.

To evaluate cognitive and attentional responses across conditions, repeated measures ANOVAs are used with **Condition** (Neutral, Generic Aversive, Symmetry, Checking, Hoarding, Washing) as a within-subject factor, and **Group** (OCD, control) as a between-subject factor. Planned contrasts are conducted for each participant’s symptom-relevant image category, comparing it to both Neutral and Generic Aversive conditions. These comparisons enable the detection of symptom-specific deviations in performance beyond general emotional interference.

Correlational analyses are used to explore dimensional relationships between trait-level variables and task performance. Pearson’s *r* correlations (Bonferroni-adjusted) are calculated between SSRT and eye-tracking metrics (e.g., dwell time, first fixation latency) and individual scores on the STAI-Y2, BIS-11, and OCI-R scales.

A symptom-targeted analysis is conducted by mapping each participant’s OCI-R subscale scores to their corresponding MOCSS stimulus category (e.g., Symmetry score with Symmetry images). Mean SSRTs are then correlated with the respective symptom dimension to examine the relationship between inhibitory control deficits and specific obsessive–compulsive symptomatology. This design allows a more ecologically valid and clinically meaningful assessment of cognitive impairments within the OCD group.

Effect sizes are reported as **partial eta squared (η²)** for ANOVA models and **Cohen’s d** for planned comparisons. Statistical significance is set at **α** = **0.05**.

### Quality control and troubleshooting

Drift exceeding 1° triggers recalibration; persistent Stop success rates outside 40–60 % suggest RT instability—rectified by enlarging the staircase step to 75 ms or extending the practice block. USB polling latency is tested periodically with a loopback cable; latencies >5 ms necessitate using a motherboard USB port rather than a hub.

### Session timing and logistics

**Table:** 

Activity	Approx. duration

Consent + questionnaires	10 min
Setup & calibration	5 min
Practice block (40 trials)	4 min
MESST (6 × 100 trials, incl. breaks)	35 min
Post-STAI + debrief	5 min

### Resource availability

All raw Experiment Builder output files (.xlsx), together with a detailed, step-by-step Python notebook for data processing and post-processing, are publicly available under a CC BY license at https://github.com/eLbARROS13/MESST_Files. Researchers without access to an EyeLink system can simply repoint the hardware-specific API calls in the linkwrapper.py module to their own high-frequency eye-tracker SDK, without altering the core analysis scripts.

## Method validation

To provide preliminary validation of the Modified Emotional Stop-Signal Task (MESST), we conducted a pilot study using a normative sample of 28 participants (aged 18–29 years). Given the exploratory nature of this initial validation, all OCD-specific stimuli categories (Symmetry, Checking, Hoarding, Washing) were grouped into a single “OCD-specific” category to streamline analyses.

We examined three key metrics of task performance: Stop-Signal Reaction Time (SSRT), latency to first fixation, and total dwell time. Results indicated significant differences across stimulus conditions –Stimulus 1 (Neutral) and Stimulus 2 (Generic Aversive)–, confirming the sensitivity of MESST to emotionally charged stimuli.

**Fig. 3:**
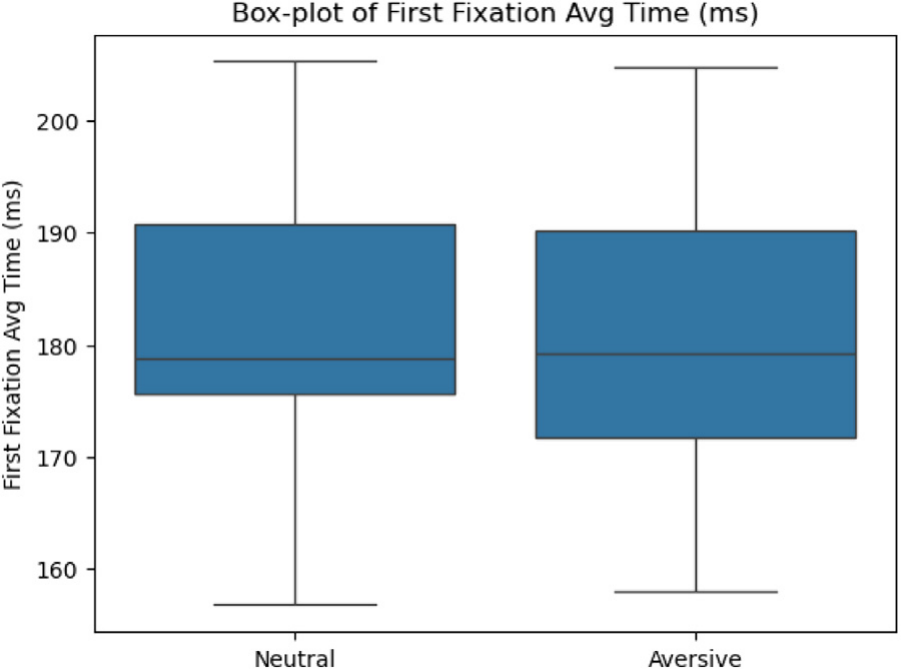
Latency to first fixation difference between S1 and S2.

**Fig. 4:**
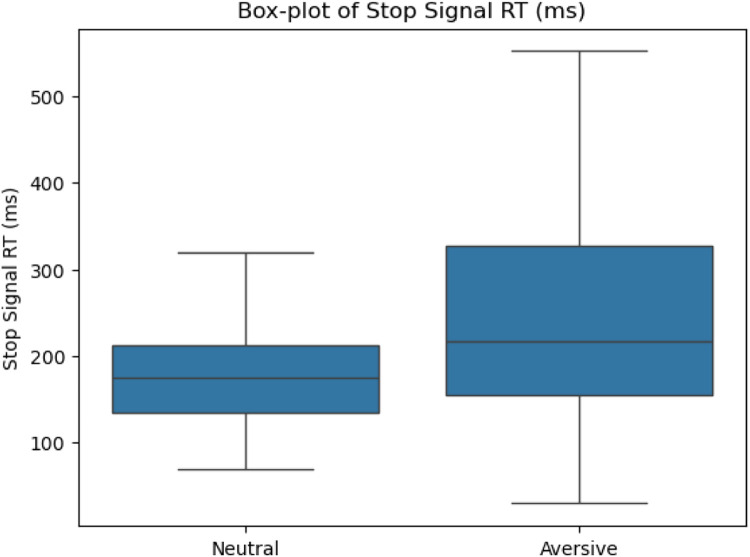
SSRTs difference between Neutral and Aversive Stimuli.

**Fig. 5:**
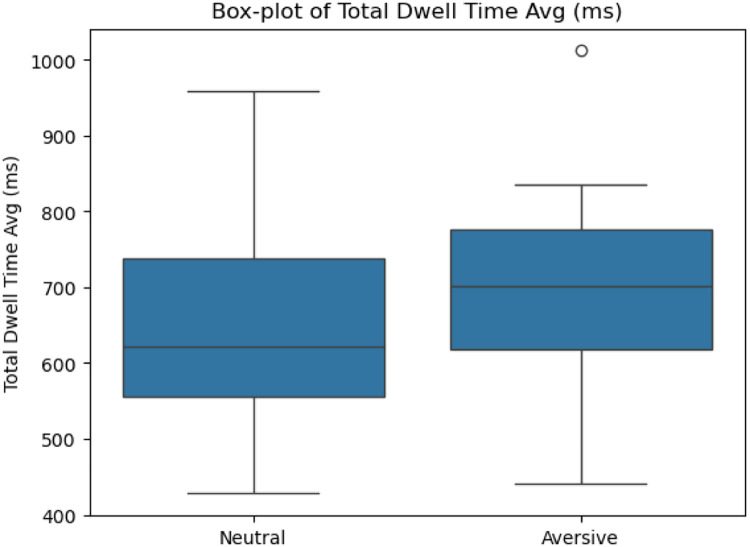
Total Dwell time difference between Neutral and Aversive.

Specifically, paired-samples *t*-tests revealed that **SSRTs** were significantly longer for Stimulus 2 (*M* = 244.23 ms, SD = 131.45 ms) compared to Stimulus 1 (*M* = 178.80 ms, SD = 65.94 ms), *t*(27) = 3.44, *p* = .002, demonstrating increased inhibitory difficulty in the presence of emotionally aversive stimuli (Fig. 4). In contrast, **latency to first fixation** was not significantly different between Stimulus 2 (*M* = 180.71 ms, SD = 12.79 ms) and Stimulus 1 (*M* = 181.13 ms, SD = 12.75 ms), *t*(32) = 0.38, *p* = .707, indicating similar attentional vigilance towards both stimulus categories (Fig. 3). Finally, **total dwell time** was significantly longer on Stimulus 2 (*M* = 701.50 ms, SD = 109.67 ms) compared to Stimulus 1 (*M* = 642.80 ms, SD = 129.64 ms), *t*(32) = −3.40, *p* = .002, reflecting prolonged attentional engagement with emotionally salient stimuli (Fig. 5).

These results align with existing evidence from Emotional Stop-Signal Tasks^21^ and eye-tracking paradigms incorporating emotional content^22^. Furthermore, they validate MESST’s capability to effectively capture inhibitory control difficulties and attentional biases triggered by emotionally relevant stimuli, supporting its future application in clinical populations, particularly individuals diagnosed with OCD.

## Limitations

Despite its strengths, the Modified Emotional Stop-Signal Task (MESST) presents some limitations:

• **Specialized equipment requirement**: MESST relies on high-frequency (2000 Hz) eye-tracking technology, requiring specialized and potentially costly equipment such as the EyeLink Portable Duo. Although systems with sampling rates ≥250 Hz—the recommended minimum for reliable detection of saccades and fixations—may support partial implementation, lower-frequency devices (<250 Hz) lack the temporal precision necessary for latency-based measures. This may hinder accurate replication of the protocol in laboratories without access to high-resolution eye-tracking systems.
• **Participant fatigue and emotional discomfort**: Due to the task length (approximately 600 trials, around 55–60 min total), participants, particularly clinical populations such as individuals with OCD, may experience fatigue or increased anxiety triggered by repeated exposure to emotionally salient stimuli. To mitigate this, experimenters should include frequent breaks (BetweenBlocksBreaks=∼0.2*BlockTime) and closely monitor participants’ comfort levels.
• **Stimulus generalizability**: While MESST employs personalized OCD-relevant stimuli to improve ecological validity, these stimuli might limit generalizability to other emotional or psychiatric contexts. Adaptation to other psychiatric conditions (e.g., PTSD or addiction) would require careful re-selection and validation of stimuli tailored to those populations.
• **Exclusion of severely symptomatic individuals**: Due to ethical and methodological considerations related to emotional distress, the MESST may not be suitable for severely symptomatic or emotionally unstable participants, potentially limiting its clinical applicability.

Awareness and proactive management of these limitations will enhance the practical utility and robustness of MESST across diverse research contexts.

## Ethics statements

The present study was conducted following the ethical standards outlined in the Declaration of Helsinki and was approved by the Ethics Committee of the Faculty of Health Sciences and Nursing of the Portuguese catholic University. Written informed consent was obtained from all participants in the preliminary validation sample prior to their inclusion. Participants were explicitly informed about their right to withdraw at any stage without any penalty or need for justification.

## CRediT authorship contribution statement

**Gonçalo Barros:** Conceptualization, Methodology, Software, Investigation, Data curation, Writing – original draft, Writing – review & editing. **Filipa Ribeiro:** Conceptualization, Writing – review & editing, Supervision.

## Declaration of competing interest

The authors declare that they have no known competing financial interests or personal relationships that could have appeared to influence the work reported in this paper.

## Data Availability

Data will be made available on request.
